# Cannabinoid Modulation of Neuroinflammatory Disorders

**DOI:** 10.2174/157015912800604515

**Published:** 2012-06

**Authors:** Viviane M Saito, Rafael M Rezende, Antonio L Teixeira

**Affiliations:** 1 Laboratory of Immunopharmacology, Graduate Program in Neurosciences, UFMG, Belo Horizonte, Brazil; 2 Laboratory of Immunobiology, Department of Biochemistry and Immunology, Institute of Biological Science, UFMG, Belo Horizonte, Brazil; 3 Neurology Group, Department of Internal Medicine, School of Medicine, UFMG, Belo Horizonte, Brazil

**Keywords:** Cannabinoid receptors, Cannabinoids, Inflammation, Multiple sclerosis, Nervous system diseases, Parkinson Disease.

## Abstract

In recent years, a growing interest has been dedicated to the study of the endocannabinoid system. The isolation of *Cannabis sativa* main psychotropic compound, Δ^9^-tetrahydrocannabinol (THC), has led to the discovery of an atypical neurotransmission system that modulates the release of other neurotransmitters and participates in many biological processes, including the cascade of inflammatory responses. In this context, cannabinoids have been studied for their possible therapeutic properties in neuroinflammatory diseases. In this review, historic and biochemical aspects of cannabinoids are discussed, as well as their function as modulators of inflammatory processes and therapeutic perspectives for neurodegenerative disorders, particularly, multiple sclerosis.

## 1 INTRODUCTION

*Cannabis sativa* is a herb belonging to the *Cannabaceae* family, characterized by palmate leaves and numerous fibers. Its first record as a medicine dates back to 5000 years ago and it was found in China, where cannabis was used for a myriad of purposes and diseases, including malaria, neuropathic pain, nausea, sexual dysfunction and constipation [1]. The use of cannabis spread from Central Asia and deeply influenced Indian folk medicine. However, sedative and psychotropic effects of cannabis turned it into a recreational drug. This fact resulted in discrimination against the consumption of the cannabis plant and its derivatives, which delayed the scientific findings in this field. 

Cannabinoid compounds may be extracted from the plant (phytocannabinoids) or be artificially obtained (synthetic cannabinoids). Mammals also produce endogenous substances (named ‘endocannabinoids’) that resemble the bioactive constituents of the plant. Since the first accurate description of a cannabis constituent in 1964 [2], enormous advances in the cannabinoid field were possible. From the discovery of Δ^9^-THC (THC), several phytocannabinoids were purified and identified, such as cannabidiol (CBD), cannabigerol, and cannabichromene. In the 1980s the presence of a cannabinoid receptor in the brain was identified by Devane and colleagues [3]. The high-affinity, stereoselective G protein-coupled cannabinoid receptor in the rat brain tissue was termed CB_1_ receptor, and soon it was cloned [4]. To CB_1_ receptor are attributed all psychotropic and motor impairing effects of cannabis, due to its plentiful expression in specific regions of the central nervous system (CNS) such as the hippocampus, pre-frontal cortex, basal ganglia and cerebellum [5, 6]. Also, because CB_1_ receptors are expressed in CNS areas related to the descending spinal inhibition, for instance, dorsal periaqueductal gray (PAG) and rostral ventrolateral medulla (RVM), they are associated to the control of pain [7-9]. In fact, synthetic THC (dronabinol) and its derivative nabilone are already marketed as therapeutic agents (Marinol® and Cesamet®, respectively) with antiemetic and analgesic properties [10-12]. Cannabinoid CB_1_ receptors are also found to a lower extent in peripheral tissues, including the adrenal gland, bone marrow, heart, lung, prostate, testis, thymus, tonsils, and spleen [13]. At a cellular level, CB_1_ receptors are found mainly at the terminals of central and peripheral neurons, where they usually modulate the release of excitatory and inhibitory neurotransmitters [14]. 

A second subtype of cannabinoid receptor, named CB_2_, was found to be expressed primarily in cells of the immune and hematopoietic systems, including myeloid, macrophage, mast, B, T, and erythroid cells [15]. Indeed, further studies on the function of this receptor identified it as the major participant in cannabinoid-mediated immune modulation [16, 17]. Additionally, the presence of CB_2_ in the CNS was also detected [18, 19], particularly in specific regions of the brain, spinal cord and dorsal root ganglia [20, 21]. Microglia cells, which are the CNS ‘macrophages’ and therefore can be considered as the resident immune cells of the brain, undoubtedly express CB_2_ receptors depending on the activated state of the cell [22-27]. Thus, microglia in healthy brain seems to not express CB_2_, whereas such receptors are detected in microglia from patients with neurodegenerative disorders and/or neuropathic pain [24, 28-30]. However, CB_2_ expression on CNS neurons is still controversial, although its presence in peripheral nociceptive neurons is responsible for modulating many types of pain [for a detailed review on this issue, see [31].

Based on the notion that an organism would not express receptors to once in life encounter their respective exogenous ligands, the existence of endogenous cannabinoid agonists needed to be considered as a fact. Thus, the first endogenous ligands (endocannabinoids) to be identified were N-arachidonoyl-ethanolamine (anandamide, AEA) and 2-arachidonylglycerol (2-AG) [32, 33], both of which are members of the eicosanoid group of cannabinoid CB_1_/CB_2_ receptor agonists. They are synthesized on demand in response to elevations of intracellular calcium levels and both function as neurotransmitters or neuromodulators, acting as retrograde synaptic messengers [34]. The biosynthetic and degradative pathways of AEA and 2-AG completed the evidence for the existence of an endocannabinoid neurotransmission system, formed by a class of receptors, its endogenous and exogenous ligands and respective metabolizing enzymes (fatty acid amide hydrolase – FAAH and monoacylglycerol lipase – MAGL), which terminate the action of endocannabinoids at the synapse [for a better review on this issue see [35, 36]. Thus, it is noteworthy to mention that endocannabinoids metabolizing enzymes constitute important targets for pharmacological interventions in order to treat/control neurodegenerative conditions as well as some types of pain [35, 37]. 

Synthetic analogs of phyto- and endocannabinoids have been developed to better explore the potential importance of this system for the development of therapeutic strategies in neurological disorders. These compounds include THC-like analogs and aminoalkylindole compounds typified by R-(+)-WIN55,212-2, an agonist for both CB_1_ and CB_2 _receptors. Other pharmacological tools to investigate the endocannabinoid system have been described, as selective antagonists (SR141716A (rimonabant), LY320135, SR144528) [38], inverse agonists (AM251, AM630) and as described above, inhibitors of the hydrolytic enzymes that degrade endocannabinoids (URB597, PF-750, JZL184).

Therefore, although solid efforts have been made in order to provide new insights into the biological actions of the endocannabinoid system, many questions remain yet unsolved. One of them is the possible existence of a third cannabinoid receptor, which could explain the bimodal and contradictory effects induced by cannabinoids in experimental studies. Thus, understanding the role of cannabinoids in homeostatic and/or pathological processes would favor their use as a therapeutic target for inflammatory diseases. 

## 2 CANNABINOID TRANSMISSION AND RECEPTOR PHARMACOLOGY

Due to the structure of the CB_1_ and CB_2_ receptors, they were grouped in the superfamily of G-protein coupled receptors [15]. Both receptors inhibit the adenylyl cyclase and activate mitogen-activated protein kinases (MAPKs) by signaling through Gi/o proteins. Additionally, CB_1_ receptors can also mediate the activation of A-type and inwardly rectifying potassium currents and inhibition of N- and P/Q-type calcium currents [14]. Unlike the CB_1_ subtype, CB_2_ receptors neither modulate the activity of P/Q-type calcium channels nor the influx of potassium [39]. Rather, intracellular CB_2_-dependent signaling stimulates MAPKs and phosphoinositide 3-kinase pathways, besides leading to the *de novo* ceramide synthesis and to the cyclooxygenase-2 (COX-2) expression [40]. 

It is noteworthy to mention that the receptor response to a specific cannabinoid depends on the ligand concentration, the presence of other cannabinoid ligands, the receptor density and its state of activation as well as the levels of signaling proteins [41-45]. Other cannabinoid effects may be mediated by non-CB_1_/CB_2_ receptors, as discussed elsewhere [14, 46].

## 3 EVIDENCE FOR THE PARTICIPATION OF CANNABINOIDS IN NEUROINFLAMMATORY PROCESSES

The presence of both CB_1_ and CB_2_ receptors on immune cells [13], and the evidence that cannabinoids inhibit adenylyl cyclase in such cells through a pertussis toxin-sensitive mode, first suggested a role for cannabinoid receptors in the modulation of the immune system [15]. Moreover, activation of immune cells by a range of inflammatory stimuli modulates the expression of CB_1_ and CB_2_ by these cells, a fact that has been linked to the immune-regulatory effects of cannabinoids [20, 47]. *In vitro* experiments have reported that cannabinoids may act as immunomodulators by (1) induction of apoptosis, (2) inhibition of cell proliferation as well as cytokine and chemokine production, and (3) expansion of regulatory T cells [48, 49]. In addition, there is also evidence of endocannabinoid production as metabolites of activated immune cells [50, 51]. 

It has been reported that cannabinoids suppress the production of a variety of pro-inflammatory cytokines in both human cell cultures and animal models, an effect that has been thought to be mediated mainly by CB_2_ receptors [52, 53]. When activated, CB_2_ receptors can modulate immune cell migration and cytokine release both outside and within the brain [54]. Furthermore, through CB_2_ receptors, cannabinoids may inhibit the production of tumor necrosis factor (TNF)-α, interleukin (IL)-1β and the p40 subunit of IL-12 and IL-23 by microglia and macrophages [55-57]. Moreover, CB_2_ receptors also seem to play an important role in regulating cell migration of neutrophils, macrophages, NK and B cells [20]. Also, it has been suggested that cannabinoids are able to decrease IL-2 production and T cells proliferation [20] and may alter the cytokine profile from a T helper (Th)1 to a Th2 phenotype in a CB_2_-dependent manner [53, 58]. However, CB_1_ receptors have also been related to neuroinflammation regulation. Notably are the data from Mestre *et al.* [59], showing that anandamide, through CB_1_ receptor activation, inhibits the expression of Theiler’s virus-induced vascular cell adhesion molecule (VCAM)-1, an endothelial receptor that plays a key role in leukocyte transmigration in multiple sclerosis [60, 61]. Zoppi *et al.* [62] also demonstrated that CB_1_ receptors activation by a synthetic selective CB_1_ agonist (arachidonyl-2'-chloroethylamide; ACEA) modulates stress-induced conditions and neuroinflammation by preventing the decrease in glutamate uptake and glutamate astroglial transporter excitatory amino acid transporter 2 (EAAT2) expression, the increase in pro-inflammatory molecules (cytokines, nuclear factor kappa B – NF-κB) and enzymatic sources, such as inducible nitric oxide synthase (NOS-2) and cyclooxygenase-2 (COX-2), in addition to the increase in lipid peroxidation.

Toll-like receptors (TLRs), considered as pattern recognition receptors (PRRs) that recognize molecular signatures of microbes known as pathogen-associated molecular patterns (PAMPs) have been emerged as central keys in the innate immune and neuroimmune responses [63-64]. Indeed, TLRs have been found to be expressed on glial cells and neurons [65-70]. Such receptors after being activated by PAMPs lead to intracellular signaling cascades that culminate in NF-κB translocation to the nucleus and the consequent synthesis of pro-inflammatory molecules [71]. Interestingly, as discussed above, cannabinoid receptors are also present on glial cells and a large body of evidence suggests that cannabinoids (phyto-, synthetic and endocannabinoids) can negatively modulate TLR4-induced neuroinflammation in these cells [72-76]. Moreover, as recently published by Downer *et al.* [77], the synthetic cannabinoid WIN-55,212-2 inhibits the pro-inflammatory signaling axis triggered by TLR3 and TLR4, while selectively augmenting TLR3-induced expression of IFN-β. 

Thus, due to a myriad of neuro-protective, anti-neuro-inflammatory and anti-oxidant actions, cannabinoids have been cogitated as possible therapeutic agents for neurodegenerative disorders that combine inflammatory responses, as Alzheimer’s Disease (AD), Multiple Sclerosis (MS), Huntington and Parkinson Diseases [78]. Hyperactive microglia, a common feature of these neurodegenerative diseases, secrete a number of pro- and anti-inflammatory cytokines, chemokines, glutamate, prostanoids, neurotrophic factors, and a range of free radicals that provide a milieu for oxidative stress. In this context, cannabidiol (CBD) has emerged as a promising strategy to treat inflammation that results from microglial hyperactivation [78], with no psychotropic side effects. Moreover, CBD has been shown to attenuate oxidative and nitrosative stress in several human disease models [78-81]. Other cannabinoid compounds that have been used in preclinical studies are exemplified in Table **1**.

Alzheimer’s disease (AD) is characterized by the presence of beta-amyloid (Aβ) plaques and neurofibrillary tangles in the brain, as well as cognitive decline and memory deficits. The abnormal processing of a 42-amino acid peptide (Aβ peptide) that precipitates in the extra-cellular space may be the cause of Aβ plaques accumulation, which leads to neuronal death. Activated microglia are found surrounding Aβ plaques, even before neuronal death [97]. In AD patients, increased microglial CB_1_ and CB_2_ receptor expression is found especially in plaque-bearing areas [84], suggesting a role of cannabinoids in AD pathophysiology. Eubanks *et al*. [82] found that THC competitively inhibits the enzyme acetylcholinesterase (AChE) by binding to the peripheral anionic site of AChE, the critical region involved in amyloidogenesis. Other studies have been conducted to support the participation of cannabinoids in AD (see Table **1**).

Parkinson’s disease (PD) is a degenerative condition affecting dopaminergic neurotransmission in the basal ganglia resulting in hypokinesia [98]. Some of the neurodegenerative features of this disease include intracellular accumulation of misfolded proteins and Lewy bodies, oxidative stress, excitotoxicity, and neuroinflammation. The endocannabinoid system may be a therapeutic target because of its marked activity in the basal ganglia where it regulates neuro-transmitter release and motor activity [5, 99]. It has been demonstrated that in PD patients, endocannabinoid levels in the cerebrospinal fluid are increased [100]. As mentioned before, the use of cannabinoid drugs that activate CB_1_ receptors may induce motor impairment; therefore, CB_1_ antagonists are more suitable candidates as therapeutic agents in PD. Indeed, results drawn from preclinical studies have proposed that SR141716A may reduce the hypokinesia in an animal model of PD, at a low dose [95]. 

Another neurodegenerative disease involving the basal ganglia is Huntington’s disease (HD). HD has a delayed onset, defined by selective neuronal vulnerability and widespread expression of disease-related proteins during the whole lifetime. A genetic defect causes abnormal protein processing and aggregation; and cellular toxic effects involve defective autophagy-lysosomal function, transcriptional dysregulation, oxidative stress, apoptosis, mitochondrial and metabolic dysfunction [101]. HD patients develop progressive psychiatric manifestations, cognitive decline and choreiform movements [102]. In HD, there is a reduction in CB_1_ expression in the basal ganglia [87, 103], where the most prominent cell loss occurs. The downregulation of CB_1_ receptors has been thought to be a key pathogenic event in HD. Lastres-Becker and colleagues [104] found that in a rat model of HD, AEA and 2-AG levels were decreased in the striatum, while there was an increase in AEA level in the substantia nigra. These changes in endocannabinoid levels are similar to those found in the brain of HD patients. However, further studies are still necessary to clarify how cannabinoids may participate in this disorder, since many preclinical results are controversial.

## 4 PERSPECTIVES FOR MULTIPLE SCLEROSIS

One of the most promising clinical uses of cannabinoid compounds lies on the symptomatic treatment of multiple sclerosis (MS) [105], although therapies based on the expansion of regulatory T (Treg) cells have been emerged as possible targets to treat early-diagnosed autoimmune diseases [106]. MS is an autoimmune inflammatory neurodegenerative disorder characterized by nerves demyelination in the CNS. Neurological commitments mostly affect young adults (aged between 20 and 40) [16] and it is diagnosed in 30 out of 100,000 people in the world population, being women more susceptible than men. MS comprises several phenotypes (relapsing-remitting, primary progressive and secondary progressive together with less common variants) depending on the population and geographic distribution [107]. Its symptoms include weakened muscle tone, motor impairment, fatigue, numbness, vision problems and cognitive loss. However, the etiology of MS is still unknown, but epidemiological studies suggested that multiple sclerosis may occur in a genetically susceptible population who has been exposed to some environmental triggering factors, such as viral infection [16, 107]. Such factors, therefore, associated with a genetically susceptibility, drives the immune response towards myelin self-peptides which devastates the myelin sheathes, causing the neurodegenerative profile of MS. To this extent, the inflammatory-mediated damage is thought to be caused by the release of reactive oxygen and nitrogen species by immune cells, as well as that of cytokines, prostaglandins and proteases, which directly mediate cell damage [56]. 

The autoimmune characteristic of MS has been reproduced in animal models such as experimental autoimmune encephalomyelitis (EAE) and Theiler’s murine encephalomyelitis virus-induced demyelinating disease (TMEV-IDD). These models are employed in pharmacological research and provide suitable evidence for new treatment strategies. Since there is no cure for MS, any treatment is only a palliative resource. Due to its intriguing anti-inflammatory effects, cannabinoid compounds have been tested in animal models of MS as a supportive treatment for symptoms like spasticity and tremor, which are common in patients with MS (see Table **2**). Lyman *et al. *[108] demonstrated that animals pretreated with Δ^9^-THC displayed a delay in the onset of the symptoms and a reduction in severity when EAE was subsequently induced. In studies using synthetic cannabinoids, dexanabinol (HU-211) was capable to reduce inflammation and EAE score, probably by suppression of TNF-α production in the brain and peripheral blood [109]. Administration of WIN55,212-2 ameliorated the progression of clinical symptoms in mice with TMEV-IDD [105], a mouse model of chronic-progressive MS and EAE [77]. Similar results were obtained by Arévalo-Martín *et al*. [110] who found that systemic treatment with WIN55,212-2, ACEA, and JWH-015 over a period of 10 days to mice with established TMEV-IDD improved their motor function by modulating microglia and lymphocyte infiltration into the spinal cord. Interestingly, the selective CB_1_ antagonist SR141716A increased EAE clinical score probably by releasing pro-inflammatory cytokines such as IFN-γ, IL-17, IL-6, IL-1β and TNF-α in mice brain and spinal cord. However, such treatment was able to simultaneously increase CB_2_ receptors expression in brain, spinal cord and spleen of mice [111]. It is worthy of mention that combined cannabinoid medicine constituted by the two plant-derived cannabinoids THC and CBD have been formulated under an oromucosal mouth spray (Sativex®) to alleviate neuropathic pain, spasticity, overactive bladder, and other symptoms associated with MS [112].

As commented above, expansion of Treg cells has been emerged as a promising candidate therapy to treat (or even prevent) autoimmune diseases such as MS (Rezende *et al.*, manuscript in preparation [113]). It is already known that cannabinoids are able to increase Treg population, which effectively contributes to their immune-modulatory actions by dampening the uncontrolled encephalitogenic T cells proliferation and activation [48, 49, 114]. Moreover, expanding Treg cells derived from the patient itself would provide an important tool to treat/prevent MS with no side effects. 

## CONCLUSION

Due to its peculiar chemistry, cannabinoids have imposed a challenge to researchers. To date, it is still impossible to prove or rule out all benefits of cannabis described empirically by ancient herbal practitioners. For now, science aims to understand how cannabinoid compounds are associated with neuroinflammation and how cannabis-based medicine can help millions of patients worldwide. The development of safe, effective cannabis-based medicines must overcome the risk of adverse effects.

## Figures and Tables

**Table 1.** Effects of Cannabinoid Treatment for Neurodegenerative Diseases

Disease	Drug	Dose	Main Effects	Refs.
**AD**	THC	---	Inhibits acetylcholine esterase (AchE)-induced aggregation of Aβ	[82]
	Cannabidiol	2.5 or 10mg/kg, i.p., for 7 days	Reduces the transcription and expression of glial pro-inflammatory molecules in the hippocampus of an *in vivo* model of Aβ-induced neuroinflammation	[83]
	WIN55,212-2	10 µg, i.c.v., for 7 days	Prevents Aβ-induced microglial activation, cognitive impairment, and loss of neuronal markers	[84]
	SR141716A	1mg/kg, i.p., single dose	Prevents the amnesia induced by Aβ (i.c.v.) peptides	[85]
	HU210	0, 10, or 50 µg/kg, i.p., twice daily for 10 to 20 days	No effect on behavioural parameters and neuropathology in APP23/PS45 double transgenic AD model mice	[86]
**Huntington’s disease**	THC	2 mg/kg	Attenuates the motor coordination deficits of R6/2 mice on the rotarod test, ameliorates striatal atrophy and huntingtin aggregate accumulation	[87]
		10 mg/kg, i.p., daily for 8 weeks	No effect on the onset or progression of behavioral deficits in the R6/1 mouse model of HD	[88]
		n/a	Increases malonate-induced striatal lesions compared to vehicle	[89]
	SR141716A	n/a	Exacerbates malonate lesions	[89]
	HU210	0.01 mg/kg, i.p., daily for 8 weeks	Increases ubiquitin-positive protein aggregate numbers, but has no effect on the onset or progression of behavioural deficits in the R6/1 mouse model of HD	[88]
	HU308	5 mg/kg, i.p., before and after intrastriatal injection of malonate	Neuroprotection by partially reducing malonate-induced GABA deficit in the striatum and the globus pallidus of rats	[90]
	URB597	0.3 mg/kg, i.p., daily for 8 weeks	Preservation of CB_1_ receptors in the mouse striatum, but had no effect on the onset or progression of behavioural deficits in the R6/1 mouse model of HD	[88]
	WIN55,212-2	5 or 10 µM, intracerebral (striatum)	Prevents quinolinic acid-induced glutamate outflow in the striatum of rats, an effect fully banned by AM251	[91]
	Cannabidiol	Average daily oral dose of about 700 mg/day for 6 weeks	Neither symptomatically effective nor toxic, relative to placebo, in neuroleptic-free patients with HD	[92]
**Parkinson’s disease**	WIN55,212-2	4 mg/kg, i.p., for 5 days	Protects mouse nigrostriatal DA neurons from the neurodegenerative effects induced by the neurotoxin MPTP; reverses MPTP-induced motor abnormalities, inhibits MPTP-induced microglia activation	[93]
		6 mg/kg, s.c., for 14 days	Morphological and cytoskeletal changes following WIN treatment, suggesting neuronal plasticity	[94]
	SR141716A	0.1 mg/kg, i.p.	Partially attenuated the hypokinesia shown by 6-hydroxydopamine-injected rats	[95]
	THC	3 mg/kg, i.p., daily for 14 days	Prevent neuronal damage induced by 6-hydroxydopamine unilateral injection into the nigra, pars compacta, in rats with hemiparkinsonism	[96]

Abbreviations: AD: Alzheimer’s disease; Aβ: beta-amyloid; HD: Huntington’s disease; i.c.v.: intracerebroventricular; i.p.: intraperitoneal; MPTP: 1-methyl-4-phenyl-1,2,3,6- tetrahydropyridin; n/a: not available.

**Table 2.** Pre-clinical Studies using Cannabinoids on Experimental Autoimmune Encephalomyelitis and Theiler’s Murine Encephalomyelitis Virus-induced Demyelinating Disease

Disease	Drug	Main Effects	Refs.
**EAE**	THC	Delayed onset and reduced severity	[82]
	Dexanabinol	Reduction of inflammation and EAE symptoms probably due to TNF-alfa decrease in brain and peripheral blood	[83]
	WIN55,212-2	Delayed development of EAE symptoms and attenuated disease severity	[84]
	SR141716A	Increased EAE clinical score probably by increasing pro-inflammatory cytokines	[85]
**TMEV-IDD**	ACEA	Improvement of motor function by modulating microglia and lymphocyte infiltration into the spinal cord	[87]
	WIN55,212-2		
	JWH-015		

Abbreviations: EAE: experimental autoimmune encephalomyelitis. TMEV-IDD: Theiler’s murine encephalomyelitis virus-induced demyelinating disease.
